# Validation of a non-invasive, real-time, human-in-the-loop model of intracortical brain-computer interfaces

**DOI:** 10.1088/1741-2552/ac97c3

**Published:** 2022-10-18

**Authors:** Peeyush Awasthi, Tzu-Hsiang Lin, Jihye Bae, Lee E Miller, Zachary C Danziger

**Affiliations:** 1 Department of Biomedical Engineering, Florida International University, Miami, FL, United States of Amercia; 2 Department of Electrical and Computer Engineering, University of Kentucky, Lexington, KY, United States; 3 Department of Neuroscience, Physical Medicine, and Rehabilitation, Northwestern University, Chicago, IL, United States

**Keywords:** neural network, cursor control, hand, kinematics, motor cortex, motor learning

## Abstract

*Objective*. Despite the tremendous promise of invasive brain-computer interfaces (iBCIs), the associated study costs, risks, and ethical considerations limit the opportunity to develop and test the algorithms that decode neural activity into a user’s intentions. Our goal was to address this challenge by designing an iBCI model capable of testing many human subjects in closed-loop. *Approach*. We developed an iBCI model that uses artificial neural networks (ANNs) to translate human finger movements into realistic motor cortex firing patterns, which can then be decoded in real time. We call the model the joint angle BCI, or jaBCI. jaBCI allows readily recruited, healthy subjects to perform closed-loop iBCI tasks using any neural decoder, preserving subjects’ control-relevant short-latency error correction and learning dynamics. *Main results*. We validated jaBCI offline through emulated neuron firing statistics, confirming that emulated neural signals have firing rates, low-dimensional PCA geometry, and rotational jPCA dynamics that are quite similar to the actual neurons (recorded in monkey M1) on which we trained the ANN. We also tested jaBCI in closed-loop experiments, our single study examining roughly as many subjects as have been tested world-wide with iBCIs (*n* = 25). Performance was consistent with that of the paralyzed, human iBCI users with implanted intracortical electrodes. jaBCI allowed us to imitate the experimental protocols (e.g. the same velocity Kalman filter decoder and center-out task) and compute the same seven behavioral measures used in three critical studies. *Significance*. These encouraging results suggest the jaBCI’s real-time firing rate emulation is a useful means to provide statistically robust sample sizes for rapid prototyping and optimization of decoding algorithms, the study of bi-directional learning in iBCIs, and improving iBCI control.

## 1. Introduction

Intracortical brain-computer interfaces (iBCIs) use implanted electrodes to access cortical neuron activity, which is then mapped into commands for assistive devices to aid paralyzed users. Non-invasive alternatives are much less effective because they capture far fewer independent neural signals (Michel and Murray 2012, Rashid *et al* 2020), have lower spatial resolution (Stieglitz *et al* 2009, Fouad *et al* 2015, Pandarinath *et al* 2017, Willett *et al* 2017), and yield longer trial times in closed-loop cursor control than iBCIs (Waterstraat *et al* 2015, Rashid *et al* 2020). However, a fundamental iBCI challenge is translating (or ‘decoding’) the neural activity into device commands. iBCI invasiveness severely limits the number of subjects willing and able to participate in pilot studies (Kim *et al* 2008, Dekleva *et al* 2021, Stavisky *et al* 2020), which dramatically slows decoder development. Assessments of decoders using small-sample studies may overestimate how well they will perform across the entire population of potential users; therefore, the field has turned to models to develop decoders.

Models of human-operated iBCIs are available, but they have serious drawbacks. One model is to replace the human iBCI user with a monkey, which preserves the intracortical signals and closed-loop control but is still subject to severe sample size constraints and considerable training requirements (Carmena *et al* 2003, Jarosiewicz *et al* 2008, Ganguly and Carmena 2009, Ethier *et al* 2012, Kao *et al* 2017). Nor is the intact monkey a good model of a human with paralysis. Alternatively, offline neural datasets can be used to test many decoders cheaply and quickly (Wen *et al* 2021), but this approach neglects the user’s real-time error corrections and adaptations that are an essential part of learning to operate iBCIs (Kawato 1990). Consequently, offline models are not typically predictive of closed-loop iBCI performance (Chase *et al* 2009, Koyama *et al* 2010, Jarosiewicz *et al* 2013). New iBCI models that overcome the current limitations are needed.

Cunningham *et al* (2011) proposed a model to deal with these limitations, which used kinematic input recorded from healthy humans during reaching to drive a neural simulator, mirroring the structure of earlier abstract human-machine interfaces that lacked explicit neural simulators (Mosier *et al* 2005, Danziger *et al* 2009). This model successfully preserves the human user and closed-loop feedback, but it relies on two problematic simplifying approximations. First, it uses 3D hand velocity to control the velocity of a 3D cursor, which does not replicate the dimensionality imbalance in iBCIs between the high-dimensional neural input and low-dimensional device commands. Nor does it represent the differences in geometry between the abstract input space of an iBCI user’s motor imagery and the simple Euclidean geometry of the cursor motion. Second, the model generates neural firing rates by sampling exclusively from velocity cosine-tuned, independent Poisson distributions, which assumes a neural representation of kinematics that may be more easily decoded by the commonly used linear Kalman filter than are actual neural dynamics (Danziger 2014).

Here, we extend that work with a new model whose goal is to mimic the human experience of closed-loop iBCI in both neural dynamics and controllability. It uses healthy human subjects’ real time finger joint angle movements as inputs to a firing rate-generating artificial neural network (ANN) that was pre-trained on monkey intracortical and kinematic data. These emulated firing rates can be translated into device commands with any standard iBCI decoder. We call this model the ‘joint angle BCI’, or jaBCI. The jaBCI captures the input-command dimensionality and geometry imbalances of iBCIs, simulates the non-intuitive iBCI neural input space, uses data-driven networks with high representational power to emulate cortical activity, and facilitates the use of statistically rigorous human subject sample sizes. Moreover, the jaBCI can simulate the changing set of neurons recorded across subject visits and produces trial-to-trial variation in neural discharge derived from motor noise in subjects’ intrinsic finger motions (rather than sampling statistical distributions to generate artificial noise). To validate the jaBCI, we tested 25 subjects using a center-out cursor control protocol similar to that of three iBCI studies (Kim *et al* 2008 2011, Simeral *et al* 2011). We found that the emulated neural signals maintained key statistical similarities to M1 neurons recorded from monkeys and that closed-loop jaBCI performance spanning four visits was similar to that of paralyzed, human iBCI users across seven different behavioral measures. These results suggest the jaBCI can be a useful tool for iBCI decoder optimization and development.

## 2. Methods

### 2.1. Study overview

**Figure 1.**
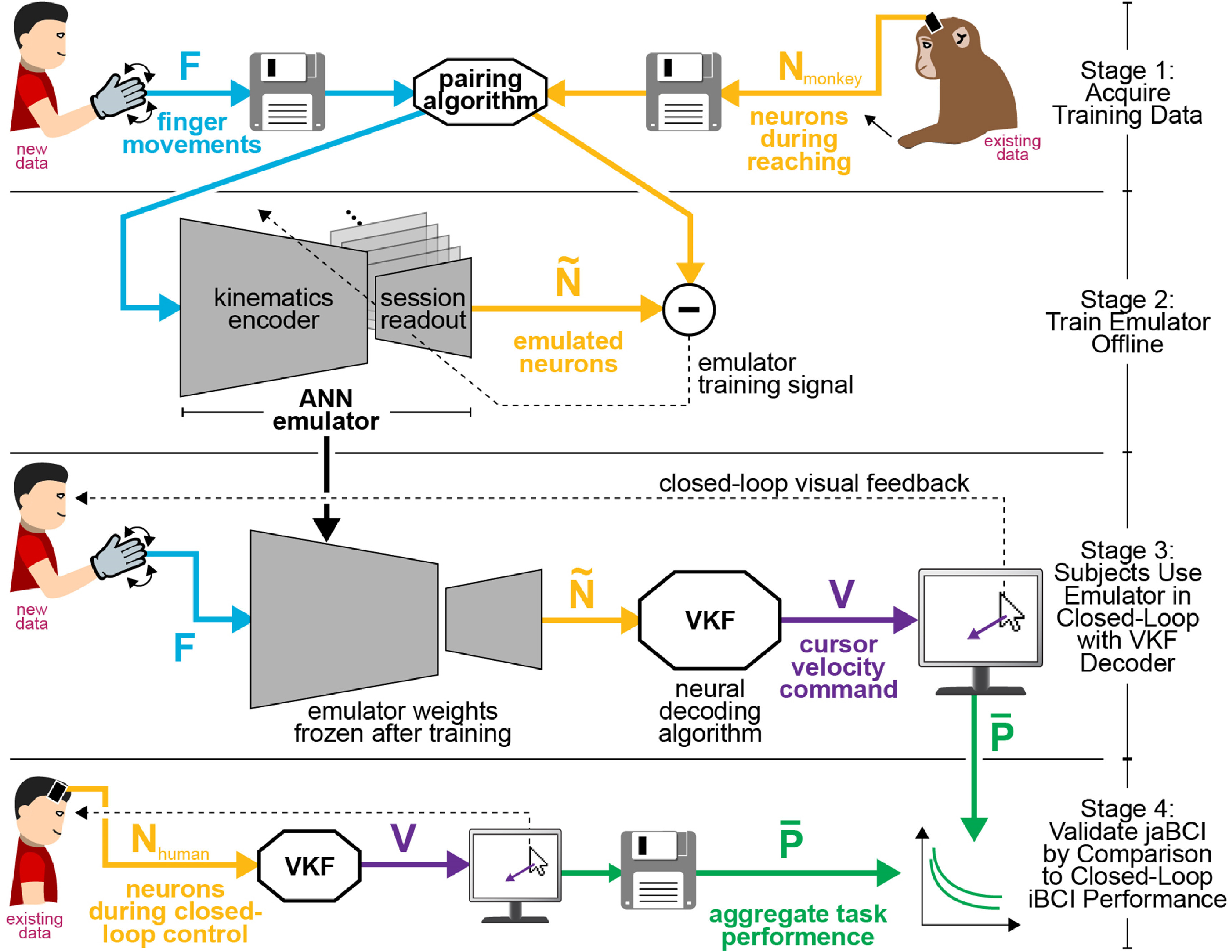
Overview of the study from data preparation to jaBCI validation. Methods subsections refer to the experimental stages shown here. Small black arrows denote physical movement, disk icons denote saved (offline) data, dashed lines denote feedback signals, and thick arrows denote multidimensional time series. Stage (1) We paired human finger kinematics with monkey neuron firing during reaching to establish an ANN training dataset. Stage (2) We trained the ANN offline to output neuron firing rates given finger movements across multiple recording sessions. Stage (3) Human subjects supplied finger movement inputs to the trained ANN to generate neuron firing, which was in turn decoded into cursor velocity, in a closed-loop target acquisition task. Stage (4) We compared the jaBCI user performance to iBCI users from prior studies using the same task and neural decoder (velocity Kalman Filter, VKF) to evaluate if closed-loop jaBCI task performance was similar to that of an iBCI.

**Figure 2.**
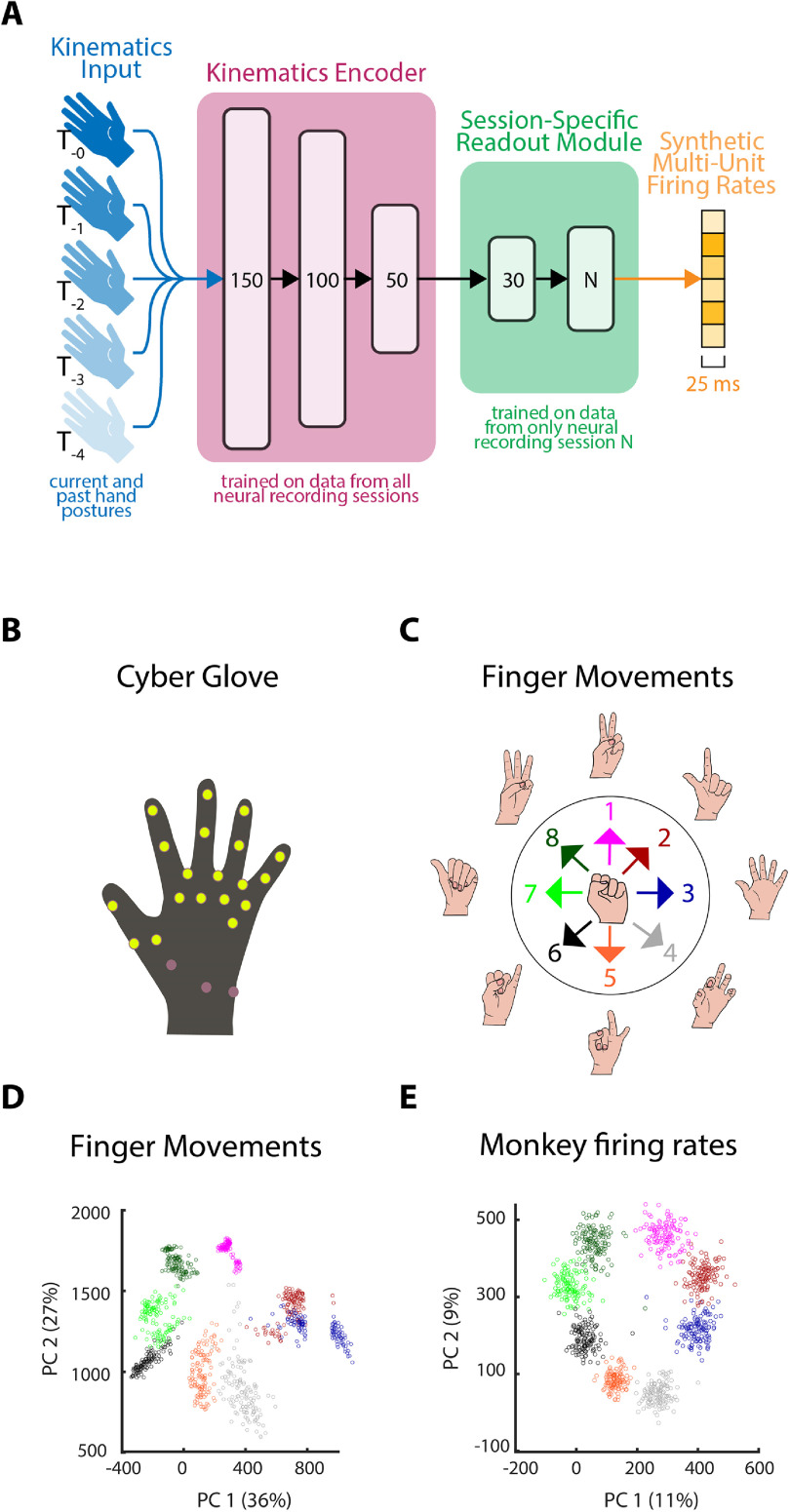
Neural emulator architecture and the paired kinematic-neural firing training data. (A) Current and time-delayed finger kinematic input vectors (blue) are represented by the hands (each a 19-dimensional vector of glove sensor values). The across-session hidden layers (pink) encoded the finger movements into a latent space. A session-specific readout module (including a hidden layer and output layer, green) mapped the latent space to firing rates associated with the neurons recorded in a particular session of monkey data. The output layers of the readout modules were different sizes, as different neurons were recorded during each session. To model neural firing rates of different sets of neurons (orange), only one session’s neurons were emulated at a time during training or closed-loop use. (B) Data glove with yellow highlighting showing the sensors used to record finger movements. (C) Illustrations of the finger movements used to generate the input training data associated with the monkey reach directions. Movements, made by the participants, started at the center posture and evolved over 1.2 s to each of the radial postures. Each transition was paired with the neural data recorded from a monkey during a reach, to produce input-output examples to train the neural emulator network. (D) PCA projection of the finger movements in panel C form a ring-like structure, where each point is colored by its movement type for that trial. (E) PCA projection of the monkey’s neural firing rates also forms a ring, with each point colored by the reach direction for that trial.

**Figure 3.**
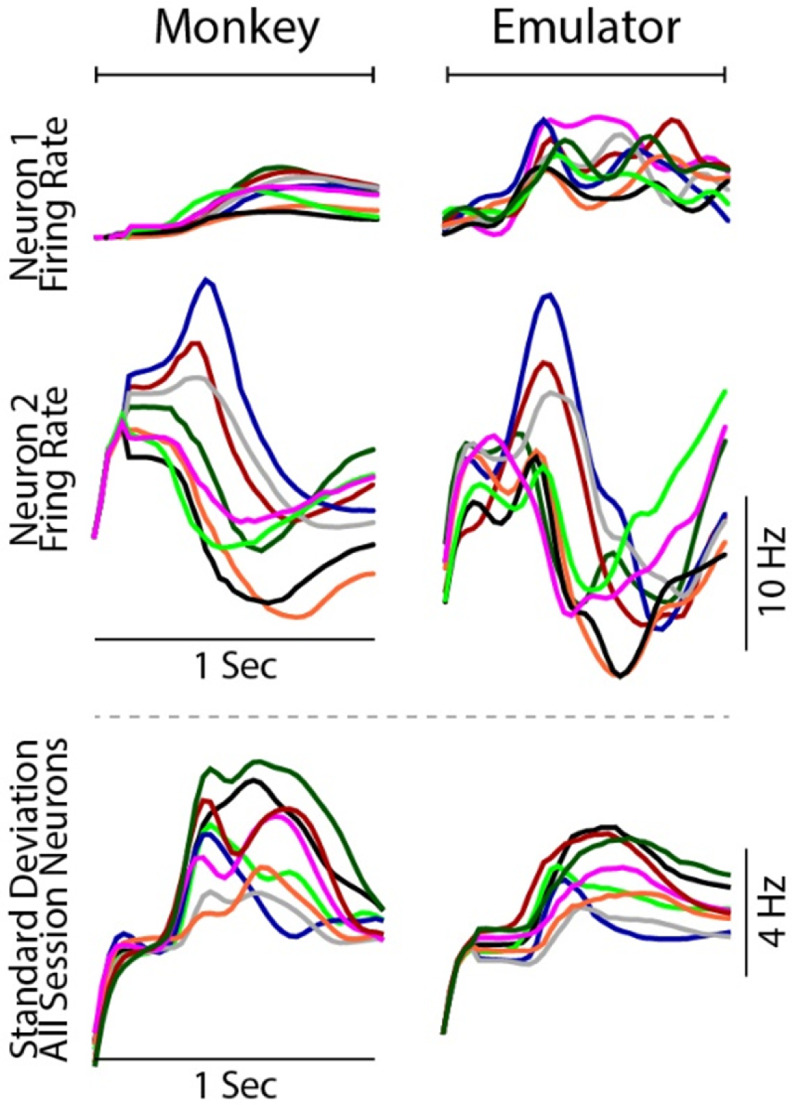
Comparison of the monkey and emulated neurons for validation trials in one session. The first and second row show example trial-averaged neuron firing rates for reaches to one of the eight directions (colors) for all validation trials in the session. Traces begin 200 ms prior to the go cue. The third row shows the standard deviation of all the individual neuron target-averages (like those shown in the first and second rows) directions. Colors correspond to target directions in figure 4, and data are from a single session.

**Figure 4.**
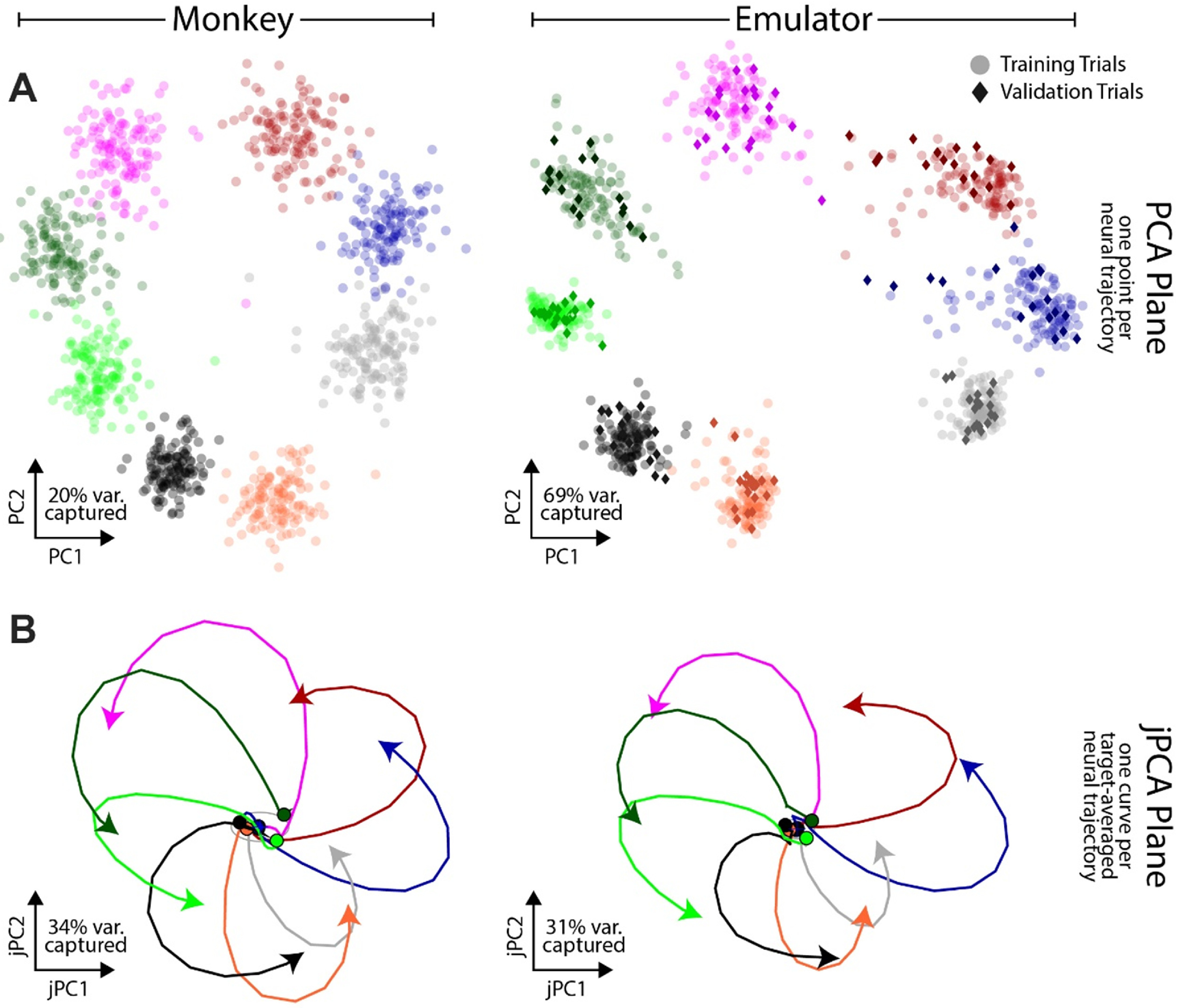
Comparison of the emulated and monkey primary motor cortex firing rate statistics. (A) PCA projection of all neurons’ firing rates for one session of monkey (left) and emulator (right), colored by monkey reach direction or finger movement, showing one point per trial. Validation trials are indicated by a diamond shape with a darker shade. (B) jPCA projection of the same session shows similarity in rotational dynamics between monkey (left) and the corresponding emulator validation trials (right), with text indicating the variance captured by the plane composed of the top two jPCs.

**Figure 5.**
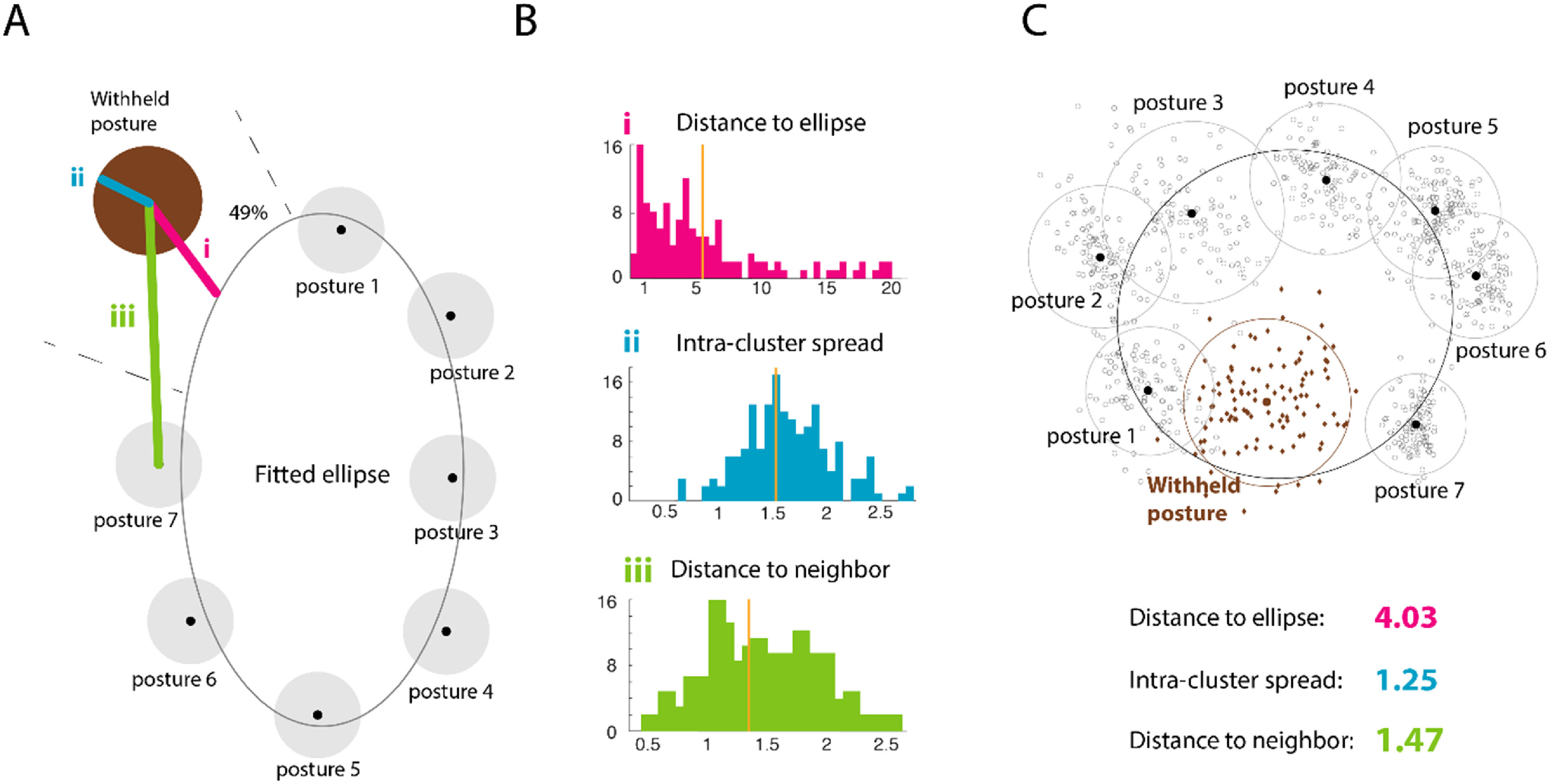
Multiple neural emulators were trained on seven of the eight training classes across all data sessions, and their firing rate outputs in response to the finger movements of the unseen class were congruent with the low-dimensional neural geometry of the seven training classes, demonstrating good generalization performance across multiple measures. (A) A schematic of the 2D PCA projection of the emulated firing rates from the reduced seven-posture neural emulators. Postures 1–7 (gray) are the training postures and posture 8 is the withheld posture (brown). (B) Histogram i (pink) shows the distance from the projected cluster center to the ellipse fitted to the training clusters, histogram ii (blue) shows the intra-cluster spread, and histogram iii (green) shows the distance to the neighboring cluster, for all 176 session-cluster instances (i.e. all combinations of eight withheld targets across 22 data sessions). The vertical lines denote distribution means. (C) An example of one reduced seven-posture neural emulator instance withholding posture 8. The training postures are in gray and the withheld posture trials are in brown. The black point in each circle represents the geometric center of the cluster. The values of each of the summary statistics for this example session are given as context to interpret the histograms in (B). The ellipse was fitted with the seven training postures.

**Figure 6.**
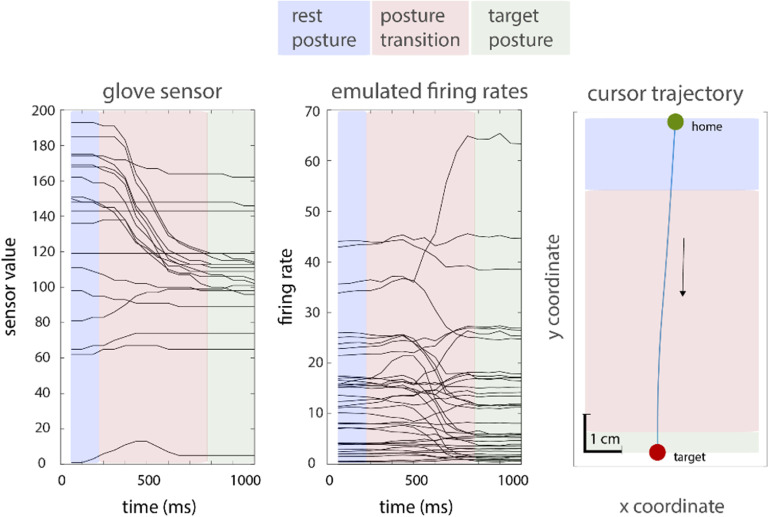
An example of a closed-loop trial. Left: finger movements, as measured by the 19 data glove sensors. Middle: emulated firing rates. Right: cursor trajectory. The participant started from a rest posture (blue), then transitioned (red) to the end in the target posture (green).

We developed and validated the jaBCI in four stages, depicted in figure 1. We indicate below, where in the methods we describe the details of each part of the model and study. The goal of the jaBCI is to use a neural emulator (an ANN, figure 2(A)) to transform finger movements of healthy human participants into synthetic neural firing rates, all in real time. To this end, in stage 1 we paired (see section 2.2.2) neural firing data recorded previously from monkey M1 during reaching (see section 2.2.1) with new finger movements from healthy subjects (see section 2.2.2) to create the ANN training dataset. In stage 2, we trained the neural emulator using the paired data and validated that its output had firing rate statistics similar to the neural training data (figures 3–5). Because the neural data included multiple experimental sessions and monkeys, we swapped out some layers of the ANN during training to account for the different neurons in each session (see section 2.2.3). In stage 3, we froze the emulator weights and had new human subjects operate the jaBCI in closed-loop (figure 6). Subjects’ finger movements were input to the emulator to generate synthetic firing rates, which were then decoded in real time to control a cursor displayed on the screen. This let subjects perform the same task (see section 2.2.1) with the same decoder (see section 2.2.2) as in a previous set of iBCI studies with human subjects. In stage 4, we compared the cursor control proficiency of jaBCI and iBCI subjects across multiple measures (see section 2.2.3). A good correspondence between closed-loop jaBCI and iBCI performance is evidence the jaBCI is a valid model for developing and evaluating closed-loop neural decoders.

### 2.2. Neural emulator

#### 2.2.1. Output training data for the neural emulator: monkey neural firing rates (stage 1)

We trained the neural emulator to output firing rates, not spike times, using data collected from two rhesus monkeys performing center-out reaches to eight targets during recording sessions on 22 different days spanning two years (Gallego *et al* 2018, Perich *et al* 2018). Data consisted of spike times recorded from discriminated single neurons in the arm area of the primary motor cortex made with microelectrode arrays from Blackrock NeuroTech (Salt Lake City Utah). Spike trains were smoothed using a Gaussian kernel of duration 100 ms to compute firing rates (Churchland *et al* 2012). The number of trials per session varied from 163 to 1037 (411 ± 311, *μ* ± *σ*). Between 23 and 93 neurons (62 ± 22) were recorded simultaneously in these sessions. Trials were segmented between 200 ms prior to and 800 ms following a go cue that signaled the reach (see (Gallego *et al* 2018, Perich *et al* 2018) for protocol details).

#### 2.2.2. Input to the neural emulator: human finger kinematics (stage 1)

We trained the neural emulator with finger kinematics input measured by a data glove with 19 sensors (CyberGlove 3, Cyber Glove System figure 2(B)) that tracked finger conformation (not hand position in space). To map the input kinematics to the output neural firing rates, we selected eight different finger movements (figure 2(C)) to correspond to the eight reach directions in the training data. The finger movements (smooth movements from the posture associated with the center in figure 2(C) to each of the postures associated with the peripheral targets) were selected to ensure they were repeatable by subjects and projected to separable clusters in a two-dimensional principal component analysis (PCA) plane (figure 2(D)) that mirrored the structure of the two-dimensional PCA projection of the neural firing rates (figure 2(E)). The selection of target postures was based on a qualitative determination of which (among the approximately 40 we investigated, largely drawn from American Sign Language) best satisfied the above criteria. We did not evaluate the sensitivity of our results to the selected postures. Each point in the projection is a finger movement starting from the initial posture and ending on a posture associated with one of the eight target directions. The projection is achieved by computing the top two principal components of the data matrix composed by concatenating the 19 glove sensor signals for one trial across columns, and stacking each trial down the rows, to create dimensions of [trials] by [sensors × timepoints]. Likewise, the neural firing rate data were projected onto the top two principal components (PCs) of the [trials] by [neurons × timepoints] data matrix.

To create a training dataset for the neural emulator, we paired finger movements with trials of neural data based on the similarity of the respective PCA geometry (described below). To generate the kinematic training data, four participants (who met our inclusion criteria but did not perform the closed-loop experiment) repeated the eight finger movements (figure 2(C)) 132 times each, once for each trial in the largest monkey dataset. The finger movements started from rest and ended at rest, allowing the emulator to mimic the corresponding monkey data at rest. We paired these kinematic inputs with neural outputs using the following algorithm: For a session of neural data we projected each trial to a point on the neural PCA plane (figure 2(E)), and each finger movement to a point on the kinematics PCA plane. With these two planes defined, we paired trials based on their relative positions in these planes. Specifically, we *z*-score normalized the 2D positions of trials separately for each target. We created a pairwise distance matrix between each kinematics and neural trial in the normalized space. We used the ‘Hungarian method’ to solve the linear sum assignment problem (Kuhn 1955), pairing kinematics and neural trials, subject to minimizing the total distance between paired trials in the normalized space. We did this pairing for each of the eight targets in all of the 22 sessions of neural data. We ran the entire pairing procedure once for each participant, thereby producing four distinct finger movement input (one per subject) for every neural output trial. The purpose of this replication step was to force the neural emulator to learn that kinematic input variation across subjects was noise, not structure to incorporate into the mapping. This reduced overfitting to the idiosyncrasies of the finger movements and improved robustness to input noise and user variation (which is high in the closed-loop human subjects experiment).

#### 2.2.3. Artificial neural network architecture and training process (stage 2)

Figure 2(A) shows the overall neural emulator architecture. We used a time delay neural network, which is a temporal association network that has previously been applied to sequence-to-sequence learning problems (Lang *et al* 1990). The temporal association is established by feeding multiple time lags of input data to the first layer. We used four time delays as well as time *T*_0_ to capture the temporal association (i.e. at time *T*_0_ the network is given *T*_0_, *T*_−1_, *T*_−2_, *T*_−3_, *T*_−4_ as the input time series). Since the goal was to use the emulator in closed-loop experiments, it effectively had one ‘bin width’ of time to compute its firing rate estimates to maintain real-time operation. Meaning, if all computations (including sampling from the data glove) were performed within the time interval used in iBCI systems to determine the average firing rates of recorded neurons, then the jaBCI model could run decoders at the same rate as iBCIs. We trained the neural emulator to compute firing rates every 25 ms because that was the shortest duration in which we could guarantee all computations would be complete with our hardware and software (and bin width choices between 25 and 100 ms show indistinguishable performance in monkey closed-loop BCI use, e.g. Cunningham *et al* 2011). The number of time lags is a meta-parameter that could be optimized heuristically. We selected four lags (100 ms of delay) based on prior work showing that optimal linear decoders perform best using 25–200 ms duration smoothing kernels (Kao *et al* 2015), Wiener filter decoders have shallow optima between 100 and 300 ms of delay (Kao *et al* 2015), and optimal characteristic times of recurrent neural network decoders are between 100 and 150 ms (Sussillo *et al* 2016). These studies suggested that 100 ms of neural history contains sufficient control-relevant information for iBCI decoders.

The input data were 95-dimensional, including 19 glove sensors × 5 current and lagged kinematic inputs. There were four fully connected hidden layers of 150, 100, 50, and 30 nodes, all with the tanh activation function. The output layer was fully connected with a rectified linear activation function. Its size was determined by the number of neurons in each recording session. Therefore, each node in the output layer was trained to match the firing rate of a single recorded neuron. All layers also had bias weights.

We used scaled conjugate gradient backpropagation to train on the loss function of mean absolute error (MAE) between the emulated and recorded firing rates (MATLAB version 2020a). We used data drawn from all recording sessions for training the ANN, but randomly split 85% of the paired trials from stage 1 into a training set, withholding 15% for validation (we used these validation data only for the PCA analysis in figure 4(A) and in selecting the initial ANN architecture). There was no need for an additional testing set because the ultimate model evaluation was determined by closed-loop jaBCI use rather than offline emulator predictions. Meaning, the goal of the jaBCI model as a whole was to create plausible neural activity when human subjects engage in closed-loop tasks with a decoder translating between the firing rates and cursor commands, not for the emulator itself to achieve high offline prediction accuracy on the monkey training set.

Motivated by the hypothesis that M1 activity acts as a relatively low-dimensional dynamical system (Churchland *et al* 2010, Kao *et al* 2015), the first three layers can be viewed as a map from kinematic input to a 50-dimensional latent space of characteristic neural activations (figure 2(A) pink). These first three layers were trained on stage 1 training data from all sessions. The last two layers were session-specific (we only updated their weights when training with the associated session of neural data, figure 2(A) green), effectively making them non-linear readout modules of the latent space representation that captured session-specific dynamics (similar to linear readout layers used in prior work (Pandarinath *et al* 2018)). This helped us avoid overfitting to stage 1 training by exposing the first three layers, which contained over 80% of the network weights, to all available training data. Therefore, the jaBCI captures the changes in neurons across days of iBCI use, but not changes that may occur within a single session of use.

To generate inter-trial neural variability when using variational autoencoders it is common to rely on randomly sampled Gaussian inputs (Wen *et al* 2021) or to predict the likelihood of neural firing, then determine spike times by sampling from Poisson distributions on the output (Cunningham *et al* 2011, Pandarinath *et al* 2018). Instead, while the jaBCI emulators were trained to generate smooth neural firing rates, when used in closed-loop in stage 3, the intrinsic variability of the user’s finger movements gives rise to inter-trial neural firing rate variation. This intrinsic noise source frees us from having to assume a particular noise distribution, or that noise channels are independent.

We used a custom neural network training schedule because the network’s transfer layer design necessitated saving and changing readout module layers throughout training (figure 2(A)). Each session of training (i.e. updating the weights of the kinematics encoder and the active readout module) was terminated when the performance gradient was smaller than 10^−6^ or after 200 iterations per trial. After the termination condition for a session, another session’s data were selected at random without replacement and we changed the readout module to that of the selected session. Training for the selected session then continued with the most recent kinematic encoder weights and the session-specific readout module weights from the most recent time it was trained. This approach amounted to a type of transfer learning (Torrey and Shavlik 2010) where one re-trains a network to handle a new instance (in this case, a neural recording session) starting with a network that was already trained on similar instances. We defined one ‘round’ as training on data from all 22 recording sessions. The training error of one round was the MAE averaged across the 22 sessions. We terminated all training when the error variance of 100 consecutive rounds converged to less than 5% of its mean (the process of changing readout modules and data sessions led to fluctuations in training performance). We selected the round with the minimum training error as the neural emulator set used for offline analysis and the closed-loop experiment.

We tuned ANN architectures and hyperparameters by grid-search and we reused the validation set to test the network variations. We searched over purely feed-forward, time-delay, and LSTM architectures, considering input delay length, hidden layer depth, layer width, and depth ratio of the kinematics encoder to the readout module. We took the 15 networks with the lowest validation loss as candidates and used the analysis in the results section 3.1 (offline analysis of the emulated neural firing rates) to manually select the final network from among the candidates.

### 2.3. Human experiment

#### 2.3.1. Closed-loop experiment protocol (stage 3)

Permission for the study was granted by the institutional review board of Florida International University. All participants signed the consent form and received compensation of $40 at the completion of their fourth visit. There were 14 males and 11 females from 20 to 41 years old who participated in this study. Each subject participated on four separate days within a ten-day interval, and the experimental protocol was identical across the four visits. We excluded subjects with neural or motor deficits impacting either hand-eye coordination or manual dexterity and subjects that had an unusually large amount of practice with tasks requiring manual dexterity (e.g. guitarists), as assessed via a self-report survey. We also excluded subjects whose hands were too large to fit comfortably in the Cyber Glove III or too small to bend the glove sensors (anecdotally estimated at less than 5% of the population). All subjects reported they had never been positive for COVID-19. We evaluated our subjects for their dominant hand dexterity at the start of their first session using the Purdue pegboard test (Brown and Jahanshahi 1998, Proud and Morris 2010).

Subjects sat comfortably with their eyes 24–30 inches from a 27” monitor (2560 × 1440 pixels) and made finger movements with their dominant hand while resting either forearm or elbow on a padded arm rest. The participants were given the opportunity to rest as needed but did not take off the data glove during the session. The workspace on the screen was 31 × 31 cm, the radius of the cursor was 1.3 cm, and targets were 5 × 5 cm with a 0.3 cm thick border.

The jaBCI modeled the daily shift in recorded neurons by randomly selecting (without replacement for a given subject) one of the 22 session-specific emulated sets of neurons (figure 2(A)). After determining the new neurons, the velocity Kalman filter was calibrated (details below). Following a set of calibration trials (see section 2.3.2), subjects used the jaBCI to perform the cursor control task taken directly from (Kim *et al* 2008) to facilitate as direct a comparison as possible with iBCI use. Exact comparisons between human iBCI studies are not possible (which further motivates the creation of a standardized human-in-the-loop model system) because each uses slightly different, fine-tuned decoder parameters, protocols, and tasks (Cunningham *et al* 2011, Danziger 2014). Briefly, each trial began with the cursor in the workspace center, which subjects attempted to pilot to one of four targets (appearing in random order) as quickly as possible before dwelling there for 500 ms to register a hit. The trial was over when the target was hit or after a 7 s time-out period. After the trial, there was an untimed period when subjects returned to the center to trigger the next trial. Subjects were free to make any finger movements. Subjects performed 80 self-paced (to avoid muscle fatigue), center-out trials on each visit. Every time the subject successfully hit the target, a gong sounded and the target turned green. The jaBCI study and Kim *et al* (2008) used the same neural decoder, dwell time, number and types of calibration blocks, time-out period, center-out task, and Fitts Index of Difficulty (we matched their initial index of difficulty of 1.2 since target sizes were changed throughout their study and we were interested in observing subjects’ initial learning rates), as summarized in table 1.

**Table 1.** Summary of protocols across three similar iBCI studies and the jaBCI behavioral study. ‘Subjects’ indicates the number of participants in each study. Subject IDs (S3 and A1) are given to the iBCI study participants to indicate which subjects were reused across studies. ‘Train Time’ refers to the duration of data used for calibrating the decoder and the ‘selector,’ if one was used in the study.

	jaBCI	Kim *et al* (2008)	Kim *et al* (2011)	Simeral *et al* (2011)
Subjects	*n* = 25	*n* = 2 S3, A1	*n* = 1 S3	*n* = 1 S3
Total No. of Subject-Visits	100	9	5	5
Decoder	Velocity Kalman Filter	Velocity Kalman Filter	Velocity Kalman Filter	Velocity Kalman Filter
Controlled Object	2D cursor	2D cursor	2D cursor	2D cursor
Bin width	25 ms	100 ms	100 ms	100 ms
Task	Center Out (4 directions)	Center Out (4 directions)	Center Out (8 directions)	Center Out (8 directions)
Train Time (min)	Decoder: 12–13	Decoder: 12–16	Decoder: 12–16 Selector: 11.6	Decoder: 6 Selector: 1
Discrete Selector (‘Click’)	None	None	Bayes Classifier	Bayes Classifier

#### 2.3.2. Decoder calibration procedure (stage 3)

We calibrated the decoder at the beginning of a subject’s visit to enable cursor control using the new randomly chosen set of simulated neurons. jaBCI users made pre-determined finger movements to generate emulated neural calibration data, which is analogous to iBCI users imagining pre-determined motor actions to generate neural calibration data. Subjects were shown either what transition to execute (jaBCI) or action to imagine (iBCI) when they see the corresponding target, which generates the data for decoder calibration.

We calibrated the decoder using the procedure described in Kim *et al* (2008). Briefly, calibration occurred after each of six 30-trial calibration blocks, taking 1.9 ± 0.2 min each. In the first two blocks, subjects watched a pre-defined straight-line cursor trajectory between targets while making finger movements over 1.5 s, each transition beginning after an audio go cue (figure 2(C)). In the final four blocks, subjects saw their controlled cursor together with the pre-defined trajectory during the finger movements. Calibration consisted in updating the measurement model, $H$, and its associated covariance, $Q$, (equation 1) in the velocity-predicting Kalman filter by linear regression.

We selected the steady-state velocity Kalman filter decoder to conform to (Kim *et al* 2008) and to facilitate comparisons to a group of iBCI studies that used similar protocols and reported similar performance outcome measures (Kim *et al* 2011, Simeral *et al* 2011). Joint angle data were sampled from the subject, converted to firing rates by the neural emulator, and decoded into cursor velocity commands once every 25 ms, creating a real-time experience like that of an iBCI user. At each iteration, *k*, the decoder mapped emulated firing rates, *z_k_*, to cursor velocity (*V_x_,V_y_*) via the Kalman measurement model (equation (1)), and the cursor position was updated by (equation (2))

\begin{equation*}{z_k} = H{x_k} + {q_k};\quad {q_k} \sim N\left( {0,Q} \right)\end{equation*}

\begin{equation*}{x_k} = A{x_{k - 1}} + {\omega _k};\quad {\omega _k} \sim N\left( {0,W} \right)\end{equation*}

$H$ and $A$ are time-invariant coefficient matrices and ${q_k}$ and ${\omega _k}$ are random vectors with zero mean Gaussian noise. The model assumes that state was linearly related to the observation ${z_k} \in {R^c}$, which represents a *C* × 1 vector containing firing rates at step $k$ for the activity of *C* observed neurons. ${R^c}$ depends on the number of neurons observed in the corresponding monkey recording session selected for use on the day of experiment.

#### 2.3.3. Data analysis of the human experiment (stage 4)

We used performance measures taken from previous iBCI studies (Kim *et al* 2008 2011, Simeral *et al* 2011, Jarosiewicz *et al* 2015) to compare closed-loop jaBCI and iBCI control:

(a) Error rate (ER): the fraction of trials in which the participant failed to reach the target within 6.5 s.
(b) Movement direction change (MDC): the number of direction changes perpendicular to the straight line joining the start position and target location.
(c) Orthogonal direction change (ODC): the number of direction changes toward or away from the start position and target location.
(d) Movement error (ME): the average distance of the subject’s cursor to the straight line between the start and the target.
(e) Movement variation (MV): the standard deviation of the orthogonal distance of each point, ${Y_i}$, on the cursor trajectory to the straight line joining start point and target (where the mean over $Y$ is defined as ${\mu _Y}$)

\begin{equation*}{\text{MV}} = \sqrt {\frac{{\mathop \sum \limits_{i = 1}^N {{({Y_i} - {\mu _Y})}^2}}}{{k - 1}}}\end{equation*}

(f) Movement time (MT): the time taken to complete one center-out trial, including the 500 ms dwell time on target to register a successful hit.
(g) Distance ratio (DR): the trajectory path length divided by the length of the straight line joining the starting and ending cursor path. The maximum possible value for DR is unity.

## 3. Results

In an iBCI, the neural discharge arising from a human user’s motor imagery is periodically decoded into device commands (Shakeel *et al* 2015, Nuyujukian *et al* 2018); analogously in the jaBCI, the emulated neural discharge arising from a healthy user’s emulator-processed finger movements is periodically decoded into device commands (figure 1). The extent to which the jaBCI accurately reflects iBCI performance-related features determines how useful this tool is to inform iBCI design. We divided jaBCI validation into two conceptual categories. The first was an assessment of the neural emulation process in isolation (stage 2 of figure 1), including the ability to reproduce firing patterns found in the validation set, reflect known dynamics in the motor cortex during reaching tasks, perform well in generalization, and be robust to small variations in the users’ inputs for a given target over trials. The second was an evaluation of the task-level performance of current subjects using the jaBCI in closed-loop compared to analogous iBCI performance taken from the literature (stage 4 of figure 1).

### 3.1. Evaluation of the neural emulator

#### 3.1.1. Average neural firing rate

To visualize the dynamics of the monkey and emulated neural activity, we computed peri-event firing rate plots of the validation trials for each of the eight reaching directions. The first and second rows of figure 3 show examples of trial-averaged firing rates for two monkey and two emulated neurons. This illustrates qualitatively the agreement in temporal characteristics for both weakly (neuron 1) and strongly responding (neuron 2) neurons. The bottom row shows the standard deviation of all monkey and emulated neuron peri-event plots on validation trials, from the same session as the individual neuron examples. These examples are qualitative evidence that the emulator mapped finger movements to firing rates which had the same gross features as the monkey neural dynamics, despite no pre-existing causal relation between them. We did this offline analysis to ensure there were no differences between high-level dynamics of the emulated and monkey neurons that would have prevented us from using the same closed-loop decoders in the jaBCI that are used in iBCI.

#### 3.1.2. PCA projection

To visualize neural activity across trials within a session, we used a planar PCA projection with a basis defined by the first two principal components for the entire session of data, such that a point represents the projection of all the neurons’ activity across one trial (details in section 2.2.2). We observed that neural activity from the monkey and emulator for any given session produced similar spatial distributions (figure 4(A)). Each point is colored by the reach direction (left) or finger movement (right). In both cases, the projected trials formed a set of eight clusters (each corresponding to one of eight directions) organized in a ring on the PCA plane. The spatial cluster ordering of the recorded data was preserved by the emulated data. The Mantel test rejected the null hypothesis that there was no correlation between the ring structure (matrix formed by the pairwise distances between the geometric cluster centers) of the monkey and emulated neural data (*p* < 0.001 for all 22 data sessions). We found strong correlations between recorded and emulated neural data clusters, averaging 0.97 ± 0.01 across the 22 sessions of data.

#### 3.1.3. jPCA projection

Target-averaged validation trial emulated firing rates exhibited similar low-dimensional, quasi-oscillatory dynamics observed in primary motor cortex during reaching (figure 4(B)). We illustrated this using the “j - principal component analysis” (jPCA) algorithm (Churchland *et al* 2012, Lebedev *et al* 2019) to decompose the recorded and emulated firing rates for each session into the six largest target-averaged rotational data modes (six chosen to be consistent with previously reported results). Figure 4(B) shows the jPCA comparison of the top two jPCs (for visualization purposes) for a typical session, illustrating that the emulator captured the rotational dynamics of the neural data. The average difference in variance explained by the top two jPCs across sessions between the monkey and emulator was less than 2% for both validation trials and training trials (see also section 4.3).

#### 3.1.4. Generalization

##### 3.1.4.1. Evaluating neural emulator predictions on novel classes

To assess the posture-to-neuron generalization, we trained multiple reduced versions of the emulator, each time withholding all trials associated with one of the eight types of training finger movements. This resulted in eight reduced seven-target emulators per session. Each reduced emulator used the same meta-parameters as the original. The response of the population of reduced emulators to inputs from the withheld classes let us evaluate if the learned map generates reasonable firing rate outputs to novel inputs, which happened frequently during closed-loop use.

To quantify ‘reasonable’, we projected the emulated neural data generated by the withheld kinematics class onto the PCA plane defined by the seven training classes and evaluated how the generalization trials clustered in relation to the training trials. We found that the cluster formed by the generalization data projected close to the ring formed by the training clusters, had comparable intra-target variability, and was near the neighboring training clusters (figure 5). The good performance of the reduced emulators suggests the architecture and training protocol leads to plausible emulated neural data in response to novel inputs. This generalization test is perhaps more rigorous than zero-shot learning since the network has no training examples or explicit contextual information about the withheld class. The following subsections describe the technical details of these measures.

##### 3.1.4.2. Quantifying emulator generalization performance

The generalization trials projected to a cluster on the PCA plane that was close to the ellipse fit to the centers of the seven training clusters (figure 5(B-i)). To ensure this measure was comparable across all reduced emulators, we normalized the generalization cluster’s Euclidean distance to the ellipse by the mean distance of the training cluster centers to the ellipse. The median of this distance was 4.0, with the distribution peaking near 1, meaning that most generalization clusters were not far from the ellipse, which is a robust result for a stringent generalization test.

The trial-to-trial variation of the generalization trials was consistent with that of the training class clusters (figure 5(B-ii)). We computed an intra-cluster variation ratio by dividing the average distance of the generalization trials to their cluster center by the average distance of the training trials to their cluster centers. The distribution of this ratio had a mean of 1.6 ± 0.5, meaning trial-to-trial generalization variability is 60% higher than for the training classes.

The generalization trials projected near their neighbor training classes (figure 5(B-iii)). For example, when we withheld posture 3, the generalization trials projected closer to training clusters 2 and 4 than the other clusters, preserving the geometrical feature of classes clustering in sequence around a ring (figure 4(A)). We calculated the average distance between the generalization cluster and its two training class neighbors and normalized it by the average distance of the all training cluster centers to their nearest neighbors (to let us compare this measure across emulators). This average distance was 1.4 ± 0.6, and 49% of the time the generalization cluster’s projection onto the ellipse fell between the appropriate training classes.

These are important generalization results because the relationship between finger kinematics and neural data does not exist independently of the training dataset, so we cannot expect *a priori* that the reduced emulators would project novel inputs to a location in PCA space that is consistent with the training classes. But the results suggest that the choice of posture training data and emulator architecture created the ability to project novel inputs congruently into the low-dimensional neural geometry created by the training class clusters. This is strong evidence that the full neural emulator learned a map that can generate realistic neural firing dynamics from novel time-varying finger movements, like those we human subjects generate when using the jaBCI in the closed-loop experiments.

### 3.2. Behavioral validations of the jaBCI

To assess the validity of the jaBCI as a tool for neural decoder design, we evaluated whether healthy human subjects using the jaBCI (*n* = 25) adopt behaviors similar to those of human users operating iBCIs with implanted, intracortical electrodes using a similar protocol (Kim *et al* 2008 2011, Simeral *et al* 2011). We used the protocol, parameter, and decoder details described in (Kim *et al* 2008), but table 1 summarizes these values for the jaBCI and the three reference iBCI studies we compared.

Figure 6 shows an example of the time-varying signals produced by a subject using the jaBCI for a single center-out trial. Colored panels show when the subject was waiting for the go-cue (blue), actively attempting to pilot the cursor (red), and dwelling on the target to register a hit (green).

**Figure 7.**
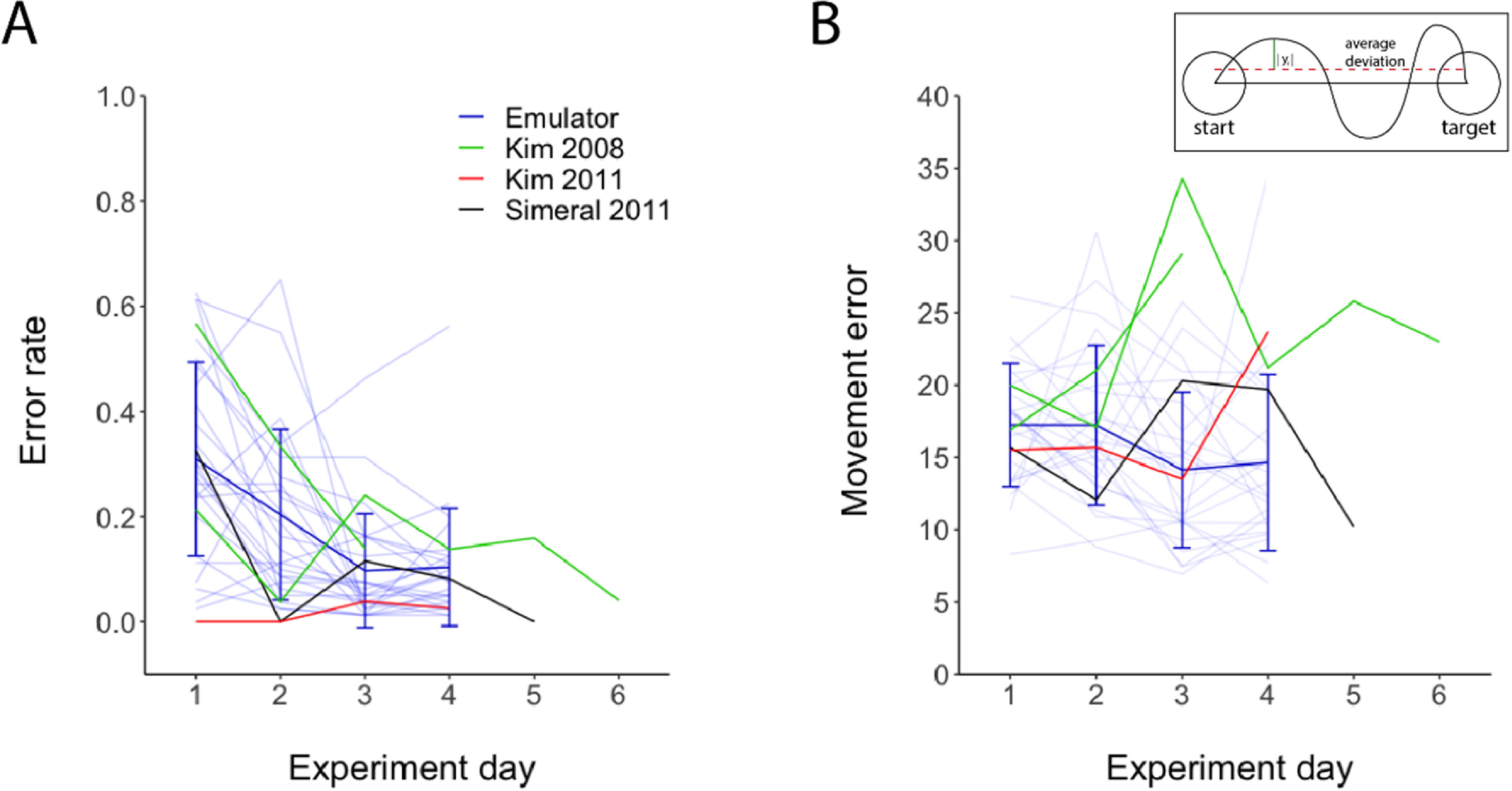
Behavioral measures of cursor control across four experimental days (return visits) of jaBCI users (25 individual subjects in light blue traces with the heavy trace showing *μ* ± *σ*) are comparable to iBCI users (green, red, black). (A) The error rate shows jaBCI subjects’ skill in acquiring the center-out targets is comparable to iBCI users, as well as their rate of improvement across visits. (B) The movement error shows that the cursor trajectories of jaBCI and iBCI users deviate from the ideal straight-line trajectory to similar extents, suggesting similar control quality.

Subjects piloting the computer cursor with the jaBCI had similar performance and cursor trajectory statistics as did subjects using iBCIs with similar protocols (figure 7). The fraction of targets subjects missed during each 80-target testing block (ER, figure 7(A)) by jaBCI subjects (blue) is consistent with the performance of iBCI subjects (green, red, black). The performance improvement trends in ER between jaBCI and iBCI users was also consistent across the four experimental days. The average cursor deviation from the straight-line path joining the starting and ending targets (ME, figure 7(B)) made by jaBCI subjects was also similar to that of iBCI users. This suggests both jaBCI and iBCI users have similar difficulty piloting the cursor straight to the target. These iBCI studies reported several additional measures of performance: cursor trajectory variance, time-to-target, and number of MDCs throughout the cursor trajectory (MDC), all of which are similar to those of jaBCI subjects. The MDC reported by (Kim *et al* 2008) was very similar to jaBCI MDC, but (Kim *et al* 2011) and (Simeral *et al* 2011) were both larger than jaBCI by 3.0 ± 1.5 (approximately 2.5 times larger), possibly due to the use of an additional linear classifier to ‘select’ targets (further highlighting the need for standardized protocols to evaluate the effects of iBCI system designs). (Simeral *et al* 2011) reported that the DR (the ratio of cursor trajectory length to straight-line distance between targets) of their iBCI user’s trajectories ranged from 1.2 to 1.9 across visits, which also agrees with jaBCI users (1.7 ± 0.7).

We found that performance measures were not affected by the particular set of neurons used in any given session (see ‘readout module’ in figure 2(A)). To quantify this, we first corrected for the effects of learning by subtracting off of each visit the average performance of all subjects for that visit (i.e. we subtracted off the learning curve across visits). We then found the average learning-corrected performance for each of the 22 sessions of neurons (note that by chance some sessions had more instances). For average target hits (max 80), the result was a standard deviation of 5.2 targets across 21 sets. There was one exception where two subjects hit approximately 30 fewer targets (5.8*σ* below the mean) than expected using a particular session of neurons. The data suggest that generally the session of neurons used does not substantively affect performance. However, we did exclude sessions that had fewer than 20 neurons from stage 1, so this result is unlikely to hold for very small sets of neurons (Wessberg *et al* 2000).

We found that the overall means of the jaBCI users’ multiple behavioral measures were correlated with each other, suggesting a more diverse set of measures could be more informative for assessing performance and trajectory quality in future iBCI studies. Measures of trajectory deviation from ideal straight-line paths, such as average MV and ME, were strongly correlated (*r* = 0.95, *p* < 0.001). Performance measures, such as mean time-to-target (MT), are also correlated with the trajectory deviation measures MV and ME (*r* = 0.78, *p* < 0.001; *r* = 0.73, *p* < 0.001), meaning larger cursor excursions are linked to longer trials. Lastly, average measures of direction changes within a trajectory orthogonal to the ideal trajectory (ODC) or parallel to it (MDC) were also correlated (*r* = 0.67, *p* = 0.001). Identifying additional, less intercorrelated measures would be more informative for assessing performance and trajectory quality in future iBCI studies.

To determine if subjects with higher manual dexterity performed better with the jaBCI, which could be a confound not present in iBCI studies, we computed the correlation between the fraction of targets subjects missed (ER, figure 7(A)) and their score on the manual dexterity Purdue pegboard test (which records how many pegs subjects can place in board slots in a fixed duration). Subjects scored between 11 and 19 pegs with an average of 13.8. There was no significant correlation (*r* = 0.17, *p* = 0.10) between pegboard test scores and ER.

## 4. Discussion

### 4.1. Overview

Motivated by the need to evaluate and optimize iBCI designs with statistically rigorous sample sizes of human users, we developed and validated the jaBCI model. jaBCI lets healthy human participants generate emulated neural signals using finger movements, and thereby operate standard closed-loop neural decoders to control external devices. The emulated neural firing rates were generated by an ANN trained offline to map finger movements to monkey cortical firing rates. The emulated neural data projected to the same low-dimensional neural geometry as the monkey data, and the emulator generated neural activity from novel classes of input data that was consistent with the structure of the monkey data. The closed-loop performance of 25 jaBCI users agreed closely with that of human iBCI users from three earlier studies who performed tasks with similar protocols and decoders (figure 7). Together, these results provide substantial evidence that the jaBCI model is a valid tool to evaluate neural decoders and user proficiency across iBCI designs.

### 4.2. jaBCI model design

Our jaBCI development efforts focused on creating a model that placed human users in closed-loop with the neural decoder to preserve the online error correction and co-adaptation dynamics that are essential to task performance (Danziger *et al* 2009, Merel *et al* 2015, Zhang *et al* 2020, Rizzoglio *et al* 2021). This avoids the problem of designing and tuning decoders offline without a clear understanding of how users will perform with them in closed-loop. Using humans also permits future studies on the initial stages of iBCI skill development and abstract cognitive tasks that are not possible when using monkeys.

Another important feature of the neural emulator is that the user’s own natural motor variability provides the jaBCI model’s noise source, in contrast to typical generative models of neural dynamics that are forced to add artificial, channel-independent, Poisson (Cunningham *et al* 2011, Churchland *et al* 2012, Pandarinath *et al* 2017) or Bernoulli (Wen *et al* 2021)—sampled noise to the model outputs to simulate inter-trial variation. The jaBCI’s biological noise source frees us from the assumptions of parametric distributions and channel independent noise when generating inter-trial variability.

We selected finger movements as the subject’s input space for multiple reasons. (a) Finger articulations are a high-dimensional source of partially independent degrees of freedom, similar to the high-dimensional and partially independent input space of motor-related neurons recorded in iBCI users (Wessberg *et al* 2000). (b) The space of finger joint angles has no obvious or natural metric, similar to the space formed by the firing rates of individual intracortical neurons, presenting both jaBCI and iBCI users with ambiguity when estimating relationships between control signals. (c) Both jaBCI and iBCI users are naive to navigating these non-intuitive input spaces, which is important for studying the rate of skill acquisition and early use co-adaptation dynamics.

The first step in creating the emulator was to link finger movements to neural data to create the neural emulator. We used human finger movements as the emulator input, instead of having our subjects make the same planar reaches the monkeys did in the training data, because 2D input is insufficient to drive the neural emulator to produce the high-dimensional neural output that is characteristic of iBCI control (e.g. see Cunningham *et al* 2011). We did not expect or require that finger movements be organized similarly to firing rates during reaching (although some similarities exist Danziger 2014); rather, the expectation was that the neural emulator would enforce the similarity by design. By training on these paired data, the neural emulator would find a transformation from finger movements to neural firing rates that were similar to those recorded during reaching (the typical motor imagery used for iBCI calibration).

The emulator training procedure does limit its scope, in part because we trained them to generate firing patterns based only on one brain region (M1). Therefore, we expect that jaBCI will be less suitable for modeling iBCIs that decode activity from different, or multiple, brain regions where we expect different neural dynamics (Lara *et al* 2018). Of course, the same approach could be taken with data from other areas as well. The neural emulators were also trained to generate firing patterns from only one task (planar reaching). Therefore, the jaBCI may be unable to model iBCIs that calibrate their decoders using very broad classes of motor imagery designed to evoke potentially many more neural patterns than were present in our training dataset (Willett *et al* 2021). This would be a more serious limitation for behaviors that are more difficult (or impossible) to imitate with monkeys. Additional work is necessary to understand if the emulator architecture described here could generate these additional patterns from suitably complex kinematic inputs.

### 4.3. jaBCI validations

We used low-dimensional projections to visualize neural activity and find Euclidean distances between clusters in PCA spaces for the emulated neural data and monkey M1 datasets. One notable difference between the stage 1 monkey data and neural emulator output was that the variance explained by the first two PCs (figure 4(A)), was lower for the monkey data than the emulated activity on the validation trials (monkey PC1 24% ± 9% and PC2 12% ± 2%, and the emulator PC1 53% ± 13% and PC2 25% ± 7%). We attribute this difference to less inter-trial variation in firing rates emulated offline, which is noticeable in the intra-cluster spread in figure 5(A), and the slightly less across-target variation (figure 3 bottom). We suspect this is a consequence of using a loss function on the mean error produced over the neural time series, which favors smoother average solutions. Supporting this hypothesis is the fact that the variance explained by the top two jPCA components of the monkey and emulated activity differed by less than 2% (pooled for training and validation trials, monkey jPC1 14% ± 5% and jPC2 16% ± 5%, and emulator jPC1 13% ± 6% and jPC2 15% ± 6%). If the emulated data corresponded to the average neural dynamics of each training class, then we expect this close jPCA agreement because jPCA computes class-averaged variance accounted for. Using a relatively low-dimensional kinematic encoding for the network may also have contributed to low inter-trial variation in the stage 1 offline data; however, the emulator did produce inter-trial differences during closed-loop use in stage 3. Presumably, that closed-loop neural variation is why subjects piloting the jaBCI-controlled cursor showed performance across multiple measures that was similar to iBCI cursor control, despite the low offline inter-trial variability.

The goal of the closed-loop study was to determine if a cohort of jaBCI users performed comparably in a cursor control task to iBCI users. Since each iBCI study uses its own fine-tuned protocols, we were unable to match any two iBCI studies exactly. We chose to replicate the protocol of (Kim *et al* 2008) because it used two naive subjects and was part of a larger three-study group using similar protocols. The heterogeneity in iBCI protocols also motivates the development of the jaBCI so that performance can be compared across many subjects while explicitly controlling for confounds such as decoders, subject re-use, and protocol details.

The jaBCI subjects improved their performance across four visits, as commonly observed during iBCI studies (Jarosiewicz *et al* 2015, Pandarinath *et al* 2017, Brandman *et al* 2018). The jaBCI performance began to plateau in the third or fourth visit; therefore, future studies examining skill acquisition using the jaBCI may not require more than four visits. The duration of the total time of center-out target trials in each session was correlated with the total duration of subjects’ untimed return to the workspace center in each session (required to trigger the next trial and advance through the protocol, *r* = 0.71, *p* < 0.001), suggesting that center-out performance is indicative of more general workspace navigation proficiency.

### 4.4. Limitations

During closed-loop jaBCI use, subjects were free to input any kinematic pattern, and we were not able to exhaustively test that all possible inputs generate plausible neural outputs. Practically, this does not appear to be a concern because the neural emulators generalized well to novel input patterns and closed-loop use was comparable to iBCI use, but we lack a theoretical guarantee.

Importantly, we lacked a way to compare the emulated neural firing during closed-loop jaBCI use directly to the monkey neural data because the human subjects did not make the same movements as did the monkeys. The tasks were necessarily different because closed-loop iBCI cursor control is less constrained and involves far more corrective actions and learning than stereotyped monkey reaches (e.g. monkey reaches last approximately 800 ms while jaBCI trials could last up to 7 s).

We constructed the neural emulator to map between causally unrelated human finger movements and monkey neural firing during a reaching task. It is an engineering tool intended to allow neurologically intact subjects to generate M1-like activity in closed-loop, not a scientific instrument to explain M1-like activity or to generate it from first principles. The goal of the emulator is to generate neural firing that would produce a decoder readout equivalent to that of neural recordings during reaching.

Lastly, we achieved sufficient similarity between emulated and monkey neural firing rates that modern decoders, which are mostly linear and have low representational power, will produce similar cursor control performance for closed-loop jaBCI and iBCI use. However, it is unclear whether nonlinear and/or highly parameterized decoders (e.g. large ANNs that are prone to overfitting) could detect and exploit differences between the emulated and biological neural firing patterns that linear or few-parameter decoders cannot. If so, we expect jaBCI results using decoders that are vulnerable to overfitting would be less likely to transfer to iBCI use.

### 4.5. Conclusions and future directions

The validated, human-in-the-loop, real-time, non-invasive jaBCI model of iBCI systems creates the possibility for conducting controlled, adequately powered studies across a wide range of potential decoder design choices. A first useful investigation using the jaBCI model is a protocol-controlled comparison of multiple neural decoding algorithms in large samples of naive subjects quantifying their performances differences. This could determine how much benefit newer iBCI elaborations confer over the traditional Kalman filters (Kim *et al* 2008 2011), such as aligning velocities to targets when training the decoder (Gilja *et al* 2012), using optimal feedback controllers (Shanechi *et al* 2016), identifying low-dimensional stable neural modes from which to decode (Farshchian *et al* 2019, Degenhart *et al* 2020), or prototyping new concepts. Similar controlled studies can be performed to determine the impact of protocol parameters like firing rate integration bin width, index of difficulty, numbers and arrangement of targets, or dwell-time versus ‘click’ selection mechanisms on human user performance. The jaBCI model could also be extended by including monkey finger kinematics paired with simultaneously recorded M1 cortical firing rates in the neural emulator training dataset. This would yield a more natural link between the closed-loop human subject finger kinematic inputs and the offline training dataset. It would also make representing recent advances in handwriting motor imagery-based iBCI control (Willett *et al* 2021) with the jaBCI more straightforward. Overall, the jaBCI is a step toward iBCI model systems that preserve the fundamental characteristics of iBCIs while allowing rapid and statistically rigorous evaluation and optimized design choices.

## Data Availability

The data that support the findings of this study are available upon reasonable request from the authors.
